# What may a fussy creature reveal about body/cell size integration under stressful conditions?

**DOI:** 10.1007/s00427-018-0613-z

**Published:** 2018-05-04

**Authors:** Aleksandra Walczyńska, Anna Maria Labecka, Mateusz Sobczyk

**Affiliations:** Institute of Environmental Sciences, Jagiellonian University, Gronostajowa 7, 30-387 Krakow, Poland

**Keywords:** Body size, Cell size, Proximate mechanisms, Stressful conditions, Temperature, Communicated by Dr. Nico Posnien

## Abstract

There is a growing amount of empirical evidence on the important role of cell size in body size adjustment in ambient or changing conditions. Though the adaptive significance of their correspondence is well understood and demonstrated, the proximate mechanisms are still in a phase of speculation. We made interesting observations on body/cell size adjustment under stressful conditions during an experiment designed for another purpose. We found that the strength of the body/cell size match is condition-dependent. Specifically, it is stronger under more stressful conditions, and it changes depending on exposure to lower temperature vs. exposure to higher temperature. The question whether these observations are of limiting or adaptive character remains open; yet, according to our results, both versions are possible but may differ in response to stress caused by too low vs. too high temperatures. Our results suggest that testing the hypotheses on body/cell size match may be a promising study system for the recent scientific dispute on the evolutionary meaning of developmental noise as opposed to phenotypic plasticity.

## Introduction

We based our study on the hypothesis that body size decreases with increasing temperature, an observation named the temperature-size rule (TSR; Atkinson 1994). It was proposed that this phenomenon is a consequence of a decrease in cell size as an adaptation to limited oxygen availability at high temperatures (Atkinson et al. 2006; Czarnoleski et al. 2015; Verberk et al. 2011; Walczyńska et al. 2015a; Woods 1999). The proximate mechanism of cell size adjustment is based on changes in the amount or structure of DNA within a cell (Czarnoleski et al. 2017; Hessen 2015; Hessen and Persson 2009; Jalal et al. 2015; Jalal et al. 2013; Kozłowski et al. 2003). DNA is mostly known from its coding and regulatory functions, but another field of knowledge is its effect on cell parameters (Gregory and Hebert 1999). This role of DNA further affects the organism body size (though not in homeotherms; Hessen 2015) and therefore has potentially substantial effects on fitness (Gregory and Hebert 1999; Gregory et al. 2000; Hessen 2015). The interrelationships between genome size, cell volume, and body size can be realized by several possible mechanisms (Cavalier-Smith 1978; Gregory et al. 2000). Curiously, the widely accepted ecological consequences of these relationships are now better understood than their proximate mechanisms (Hessen 2015; Hessen et al. 2013; Hessen and Persson 2009; Kozłowski et al. 2003).

**Fig. 1:**
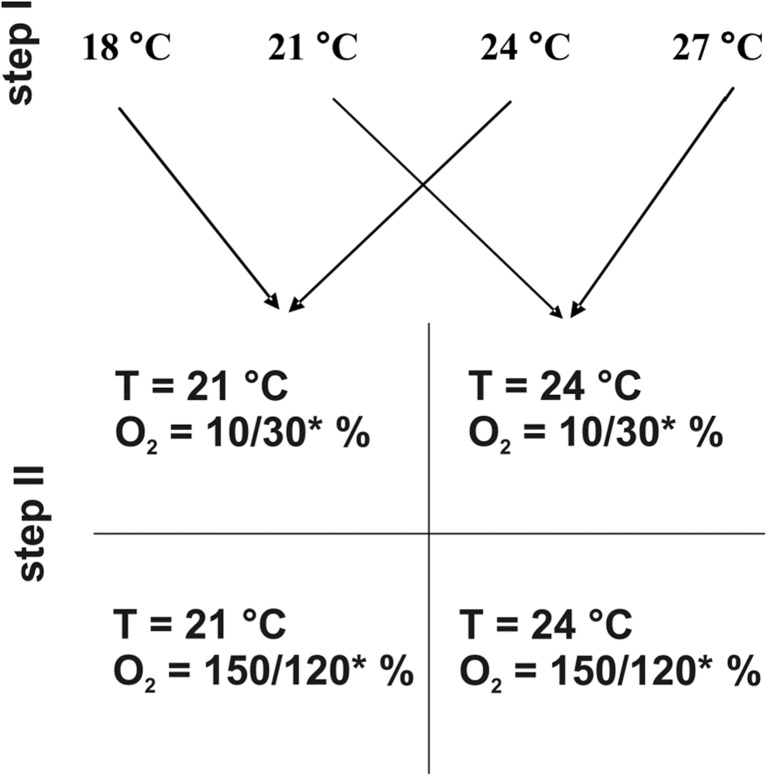
The scheme of the experiment. In step I, annelids were differentiated in body size by exposure to four different temperatures. In step II, each “size group” was placed in a combination of low/high-temperature/oxygen conditions so that the temperature change was smaller or larger by always the same amount (3 °C in this case). *The notation means the oxygen concentration in trial 1/trial 2

In this study, we analyze and discuss some interesting results on body/cell size adjustment under stressful conditions observed during an experiment designed for another purpose. We repeated the experiment using a non-eutelic annelid *Aeolosoma hemprichi* Ehrenberg 1828 on the body size effect on the population growth under low/high-temperature/oxygen conditions, which we have previously conducted on an eutelic rotifer *Lecane inermis* Bryce, 1892 (Walczyńska et al. 2015a). Both *L. inermis* (Kiełbasa et al. 2014; Walczyńska et al. 2016) and *A. hemprichi* (Walczyńska et al. 2016) were previously reported as decreasing in size with increasing temperature, following the TSR, while exposed to wide temperature range. In both a previous study on rotifers and in the current study on *A. hemprichi*, we performed a two-step study: in the first step, we aimed at differentiating the organisms’ body size by culturing them at different temperatures, while in the second stage, we examined the fitness (fecundity in rotifers and population growth rate in annelids) of the second generation of “small” and “large” individuals at combinations of low and high levels of two factors, temperature and oxygen (Fig. 1). We then related the body size changes to cell size (nucleus size in the rotifer case). In our previous study on *L. inermis*, we found support for all the hypotheses posed: body size was found to be an adaptation to temperature-dependent oxygen availability, realized through cell size adjustment (Walczyńska et al. 2015a). *A. hemprichi* appeared to be sensitive in the laboratory conditions. Due to failures at the second experimental stage, we repeated the entire experiment. Even with that, our goals were not achieved because the *A. hemprichi* population did not proliferate well in experimental treatments. However, we explored our results from the perspective of the relationship between body size and cell size under stressful conditions. We present some novel ideas regarding the match between cell and body size and its limitations.

## Methods

### Study species

*Aeolosoma hemprichi* is a 500–1500-μm-long (Menniti and Morgenroth 2010) limnetic annelid, which reproduces by paratomic fission (Falconi et al. 2006). It is a stenophagous bacterivore (Inamori et al. 1990; Ratsak and Verkuijlen 2006; Suzuki et al. 2005). The study was conducted on the *A. hemprichi* clone isolated from the activated sludge in a small wastewater treatment plant in southern Poland. Prior to this study, *A. hemprichi* was cultured at room temperature (ca. 21 °C) and natural photoperiod, without oxygen manipulation. Different sources of biofilm were tested (rice, buckwheat, and molasses), and the annelid proliferated equally well on each of them.

### Experimental design

In the first experimental stage (hereafter, step I), we aimed to induce the divergence in body size toward “small” and “large.” We cultured them in 300-mL glass containers (approximate final density of 100–200 individuals per container) with spring water (Żywiec, Poland) as a medium and three rice seeds as a biofilm source and placed them in the water baths (Memmert, Germany) set to 18, 21, 24, or 27 °C (three replicates per treatment).

In the second experimental stage (hereafter, step II), animals were exposed to four different treatments with a combination of low/high-temperature and low/high-oxygen concentration (Fig. 1). We used small plastic 5-mL tubes closed with nylon mesh on both ends immersed in water baths for that purpose. We started step II with 20 individuals of a similar medium size per replicate (three replicates per treatment). Animals were provided with the same medium and food type as in step I. To generate the experimental oxygen conditions, we used an Oxy-Reg four-channel O_2_ regulation system (Loligo Systems, Denmark). The system was calibrated for 0 and 100% of air-saturated water. Because of the problems with little animal proliferation in this second stage of the study in the first trial (trial 1), the study was repeated (trial 2). In trial 1, the step II experiment was conducted under a temperature of 21 or 24 °C and oxygen concentration of 10 or 150% (Fig. 1). The numbers of *A. hemprichi* decreased in all but one replicate after 4 days. In trial 2, the only difference in experimental design was that we narrowed the range of oxygen conditions to 30 and 120% to possibly increase the annelid survival. However, the numbers of individuals did not generally increase above the initial value after 8 days of the experiment. We decided to analyze the samples from trial 2 treating the body and cell size as a response to stressful experimental conditions. The annelid number analyses were conducted for trial 1 and trial 2 separately.

### Cell and body size measurements

**Fig. 2:**
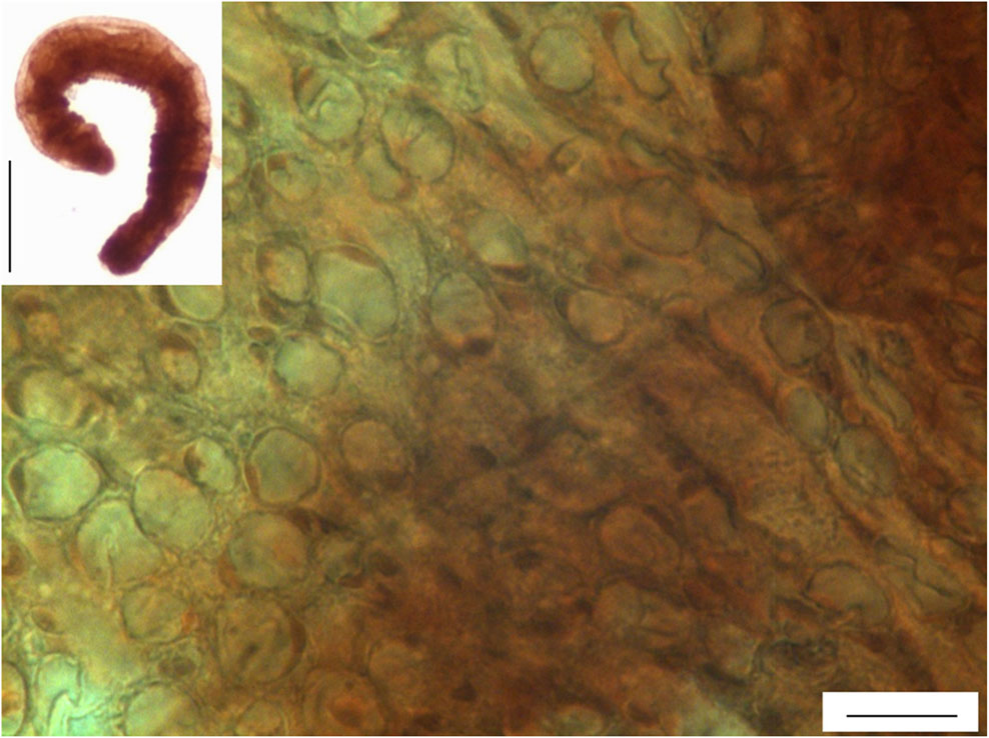
The illustration of research material used for body and cell size measurements in *A. hemprichi*. Scale bar for an annelid, 200 μm, and scale bar for white adipocytes, 10 μm

For the measurements of cell size and body size response to temperature (step I) and to temperature-oxygen conditions (step II), annelid samples were taken from trial 2 only. The annelids were fixed in 10% buffered neutral formalin (POCH, Poland). Afterward, 1 ml of 1× phosphate-buffered saline (PBS; POCH) was added, and the samples were centrifuged (2000 rpm, 2 min; Eppendorf 5702, Germany). The supernatant was replaced by fresh 1× PBS five times, and the centrifugation step was also repeated five times. Then, 1× PBS was extracted and replaced with 0.5 mL of Gill’s hematoxylin (Carl Roth, Germany). The animals were stained with hematoxylin for 4 h at room temperature and centrifuged. The *A. hemprichi* precipitate was dropped onto a glass slide and photographed under an Eclipse 80i light microscope (Nikon) equipped with an Axio CamMRc5 digital camera (Zeiss, Germany) and ZEN (Zeiss) software. The white adipocytes (unilocular cells) were chosen for cell measurements because they were the only cell type clearly visible throughout all of the animal samples. Generally, the lipid storage mechanisms in invertebrates function similarly to these of vertebrates (Azeez et al. 2014; Schlegel and Stainier 2007). The processes within adipocytes are dependent not exclusively on the amount of food but are also affected by the hormones they produce and by the nervous system (Young et al. 2006). It makes these cells possibly highly responsive to stress. They may also respond to temperature; white adipocytes in mice were found to activate thermogenesis when exposed to lower temperatures (Ye et al. 2013). Pictures of the slides were examined using ImageJ software (NIH, USA). To estimate body size, we measured perimeter as a proxy for body area (μm^2^). To assess the size of the adipocyte cells (μm^2^), we measured two perpendicular diameters of 10 cells per animal, collected from three to five photos of different body parts of the same individual. In the cases when measuring 10 cells per animal was not possible, at least five cells were measured. In two exceptional cases, only three and in one case, four cells per specimen were measured. The example of annelids and adipocytes in annelids used for measurements is presented in Fig. 2.

### Statistical analysis

The numbers of individuals surviving to the end of step II were analyzed with GLM for trial 1 and trial 2 separately, with temperature, oxygen concentration, and experienced direction of temperature change (two groups: experiencing temperature decrease (hereafter, Temp−) or increase (hereafter, Temp+) in comparison with conditions in step I) as fixed factors. The temperature effects on body size and cell size in step 1 of trial 2 only were analyzed with the Kruskal-Wallis test (an assumption on the homogeneity of variance for ANOVA was not met in the case of body size, and an assumption on the normality of residuals was not met for cell size data). Body and cell size differences in step II of trial 2 were analyzed using either ANOVA with temperature and experienced direction of temperature change as fixed factors, or ANCOVA, using cell size as a covariate. The model was tested for the possible significance of the interactions between the main factors and a covariate to check whether the assumption on the similar slope of the relationship between the dependent variable and a covariate across treatments was not violated. The relationship between body size and cell size, for both step I and step II in trial 2, were analyzed using simple regression. All the analyses were performed in Statistica 12 (StatSoft 2014).

## Results and discussion

### The effect of conditions in step II on annelid number in trials 1 and 2

**Fig. 3:**
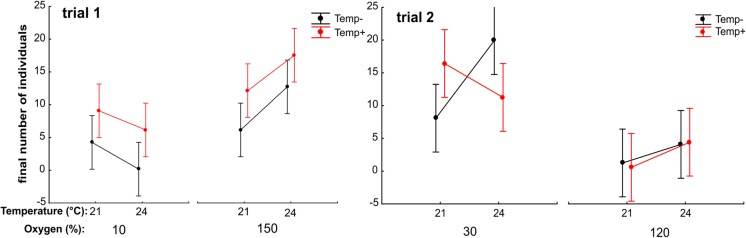
The three-level interaction effect on *A. hemprichi* numbers after the completion of step II of trial 1 (not significant) and trial 2 (significant). LS means ± 0.95 CI for the three replicates. Temp− are individuals experiencing temperature decrease in step II; Temp+ are individuals experiencing temperature increase in step II compared to conditions in step I

A new interesting observation was a significant effect of the experienced direction of temperature change, whether deriving from the low temperature in step I and experiencing the temperature increase in step II (Temp+) or deriving from the high temperature in step I and experiencing the temperature decrease in step II (Temp−). In trial 1, Temp+ annelids performed significantly better than Temp− annelids (Table 1 and Fig. 3). In trial 2, there was no difference in performance between these two groups, but this effect might have been masked by the significant interaction of temperature, oxygen, and experienced direction of temperature change in this trial (Table 1 and Fig. 3). Generally, the response of Temp+ and Temp− annelids differed despite them not differing in size in step I.

**Table 1:**
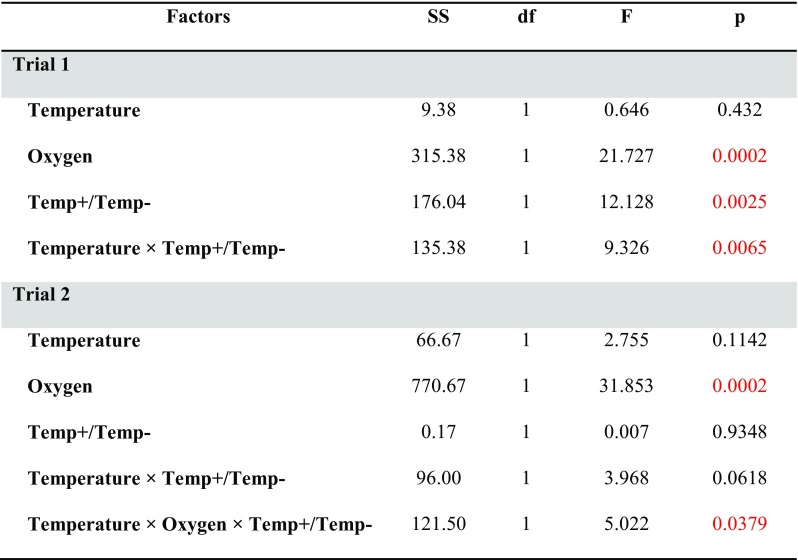
The results of GLM analysis on number of individuals of *A. hemprichi* observed after the completion of step II in trials 1 and 2

All of the non-significant interactions were removed from the models. Temp+/Temp− denotes individuals deriving from the lower temperature in step I and experiencing temperature increase in step II/deriving from the higher temperature and experiencing temperature decrease in step II. Significant effects are marked in red

**Fig. 4:**
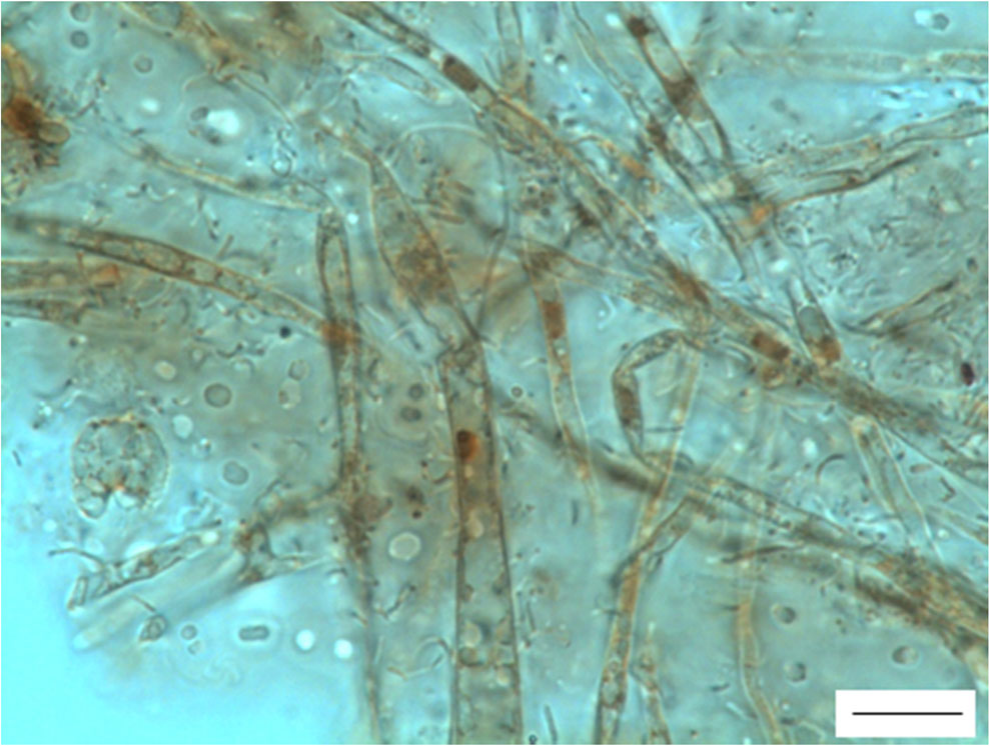
The unidentified filaments most likely disturbing the proliferation of annelids in the high oxygen concentration treatment (120%) of step II of trial 2

There was no effect of temperature on the number of annelids, while the effect of oxygen was significant, and yet, the direction of response toward low or high oxygen levels differed between trials (Table 1). In trial 1, the annelids performed better at a higher (150%) rather than at a lower (10%) oxygen level, especially at higher temperature (Fig. 3). Annelids performed extremely poorly at a high oxygen level (120%) and were in relatively good condition at a low-oxygen treatment (30%; Fig. 3) in trial 2. The most likely reason for such a difference was the presence of unidentified filamentous microorganisms (Fig. 4), which infected the samples at the 120% O_2_ treatment in trial 2. The means in which the filaments could have affected the annelids remain uncertain. The identification of these microorganisms, whether of bacterial or fungal origin, would shed more light on this issue; we assume that bacteria could have deprived the annelids of food (filamentous bacteria, not accessible to annelids, could outcompete other bacteria), while the possible effect of fungi on *A. hemprichi* could be the movement disturbance. The generally low numbers of individuals counted after the completion of trials 1 and 2 show the considerable sensitivity of *A. hemprichi* to experimental conditions. These problems were apparently caused by the sensitivity to rapid changes in temperature/oxygen conditions because annelids proliferated successfully both in a laboratory culture on various substrates and in step I of this study.

The appearance of the unknown filamentous contamination in the 120% oxygen treatment in trial 2 prevented us from obtaining unambiguous conclusions on the effect of oxygen on *A. hemprichi*. However, by comparing trials 1 and 2, we may speculate that *A. hemprichi* is sensitive to very low oxygen levels (10% in trial 1) and performs similarly well in 30 and 150% O_2_. The only difference between these two treatments was the poor proliferation of Temp+ annelids at 24 °C under 30% O_2_ compared to 150% O_2_ (Fig. 3). This result has important implications: the temperature of 24 °C is above the optimum regarding *A. hemprichi* performance measured to be 21 °C by Walczyńska et al. (2016), but such conditions seem to be limiting only under hypoxia and not hyperoxia. The joint limiting conditions of high temperature and hypoxia were previously observed during examination of fecundity in the *L. inermis* rotifer (Walczyńska et al. 2015a). From the perspective of the global warming issue, both the previous and current results validate the suggestion that it is not temperature itself, but rather its relationship with oxygen relative concentration, that matters as a factor responsible for size response (Atkinson et al. 2006; Walczyńska and Sobczyk 2017).

### The effect of temperature on body and cell size in step I of trial 2

The mean coefficient of variation (CV) for cell size measured within a specimen for individuals deriving from 18, 21, 24, and 27 °C were 26.2% (*N* = 24), 29.3% (*N* = 30), 24.8% (*N* = 21), and 27.5% (*N* = 49), respectively. The relatively high variability of adipocyte size within an animal may result from the fact that they were measured from three to five photos representing different parts of the body, while this cell type shows considerable size variability in general (Sawicki and Malejczyk 2012, for the case of humans). Yet, our approach made the results representative for an individual.

**Fig. 5:**
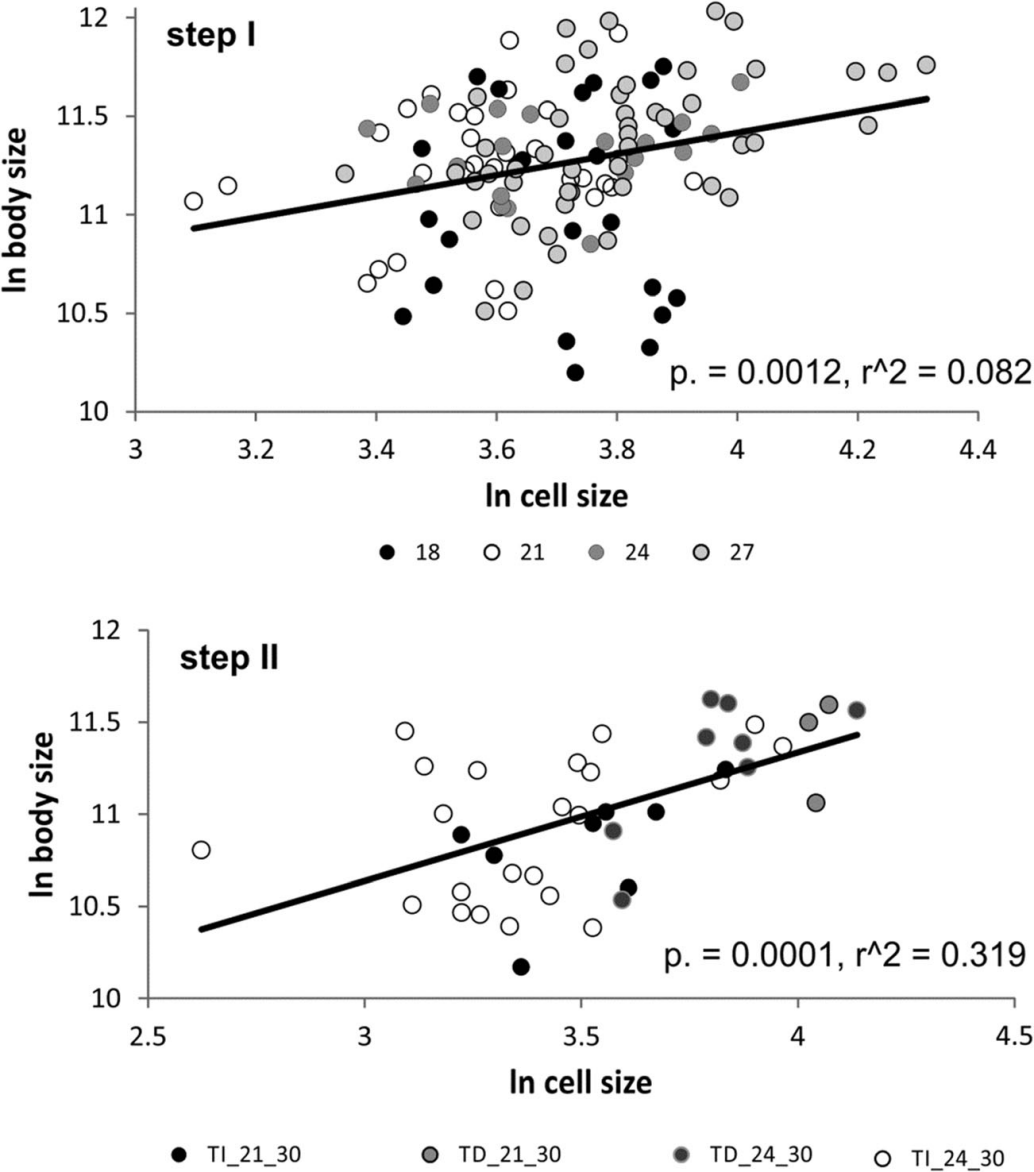
The relationship between body size and cell size in *A. hemprichi* in step I (*upper panel*) and step II (*lower panel*) conditions (trial 2). Different treatments are marked with different symbol colors

Body size did not differ across treatments (Kruskal-Wallis *H*_(3, 131)_ = 6.75, *p* = 0.0803), while cell size differed (Kruskal-Wallis *H*_(3, 124)_ = 21.64, *p* = 0.0001); cells of annelids from 21 °C were significantly smaller than those from 24 and 27 °C. In light of the previous studies on *A. hemprichi* body size response to temperature, the lack of differences in body size obtained in the present study might be caused by the chosen thermal range, which was too close to the approximate optimal value, estimated to be 21 °C (Walczyńska et al. 2016). Body size significantly depended on cell size (*N* = 124, *p* = 0.0012, *r*^2^ = 0.0823; Fig. 5, upper panel) for the data pooled for all thermal regimes.

### The effect of experimental conditions on body and cell size in step II of trial 2

**Fig. 6:**
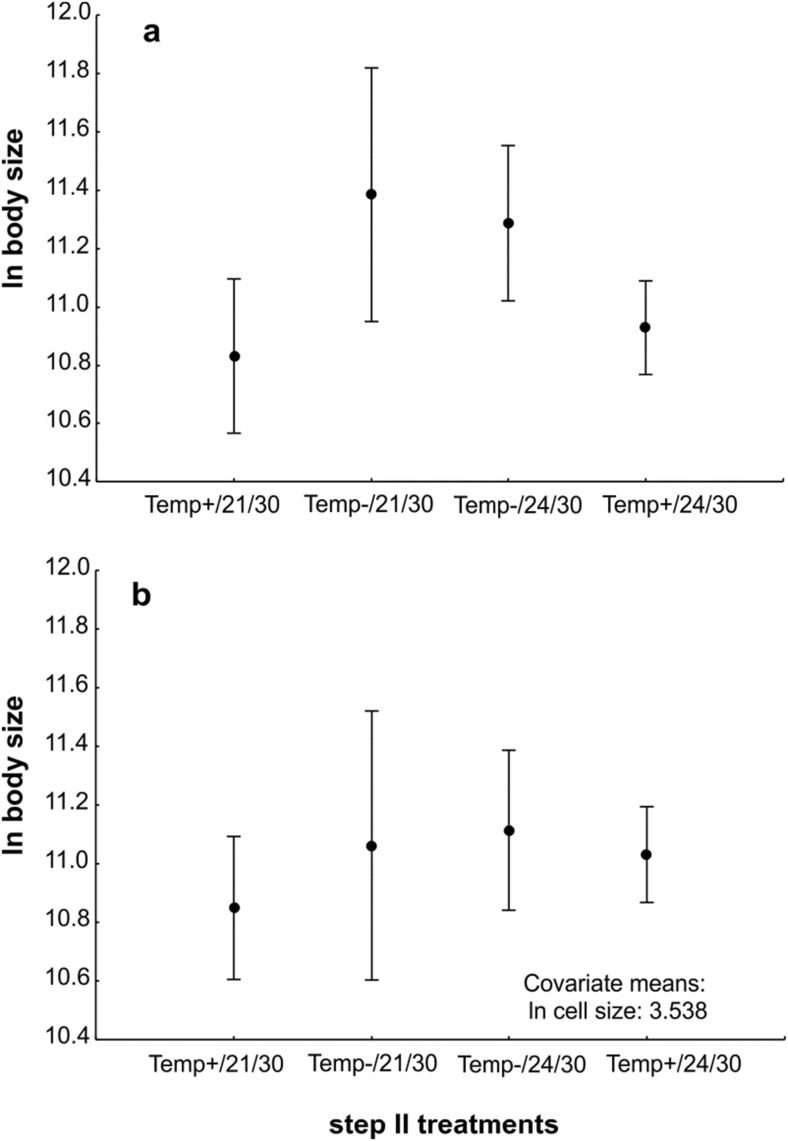
The difference in body size of *A. hemprichi* in step II treatments after simple ANOVA (**a**) and after ANCOVA with cell size as a covariate (**b**). Means ± CI. The treatment code is as follows: experienced direction of temperature change/temperature/oxygen. Temp− are individuals experiencing temperature decrease in step II; Temp+ are individuals experiencing temperature increase in step II, compared to conditions in step I

After excluding the samples from 120% O_2_ infected by filaments, the results show no effect of temperature but a significant effect of the experienced direction of temperature change (Temp+/Temp−) on body size (Table 2 and Fig. 6a). Qualitatively similar results were obtained for cell size (Table 2). A mean CV for cell size measured within a specimen for individuals deriving from treatments Temp+/21/30, Temp+/24/30, Temp−/21/30, and Temp−/24/30 were 24.5% (*N* = 8), 35% (*N* = 22), 16% (*N* = 3), and 22.6% (*N* = 8), respectively. Interestingly, including cell size as a covariate in the model for body size differences resulted in an observation that initial conditions did not have a significant effect on cell size (Table 2 and Fig. 6b); the annelids exposed to lower experimental temperature (Temp−) were not larger than Temp+ when the size of their cells was taken into consideration (Fig. 6). We explore the important consequences of this result below. Body size was significantly dependent on cell size for the data pooled for all experimental treatments (*N* = 41, *p* = 0.0001, *r*^2^ = 0.3190; Fig. 5, lower panel).

**Table 2:**
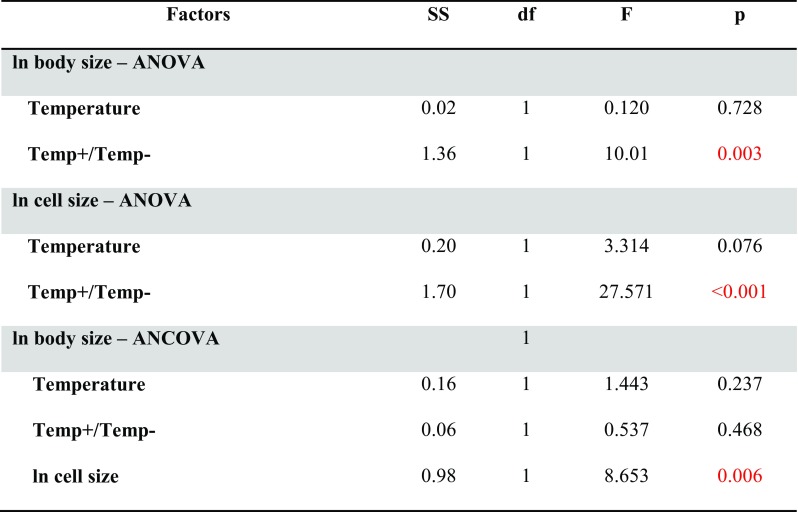
The results of GLM analysis on body and cell size of *A. hemprichi* after step II of trial 2

Significant effects are marked in red. Temp+ are individuals experiencing temperature increase in step II, and Temp− are individuals experiencing temperature decrease in step II, compared to conditions in step I

Our results inspired us to reflect more broadly on the general relationship between the cell and body size adjustment under stressful conditions. We now invoke some possible points for discussion.

### To what extent do cell size and body size correspond?

**Fig. 7:**
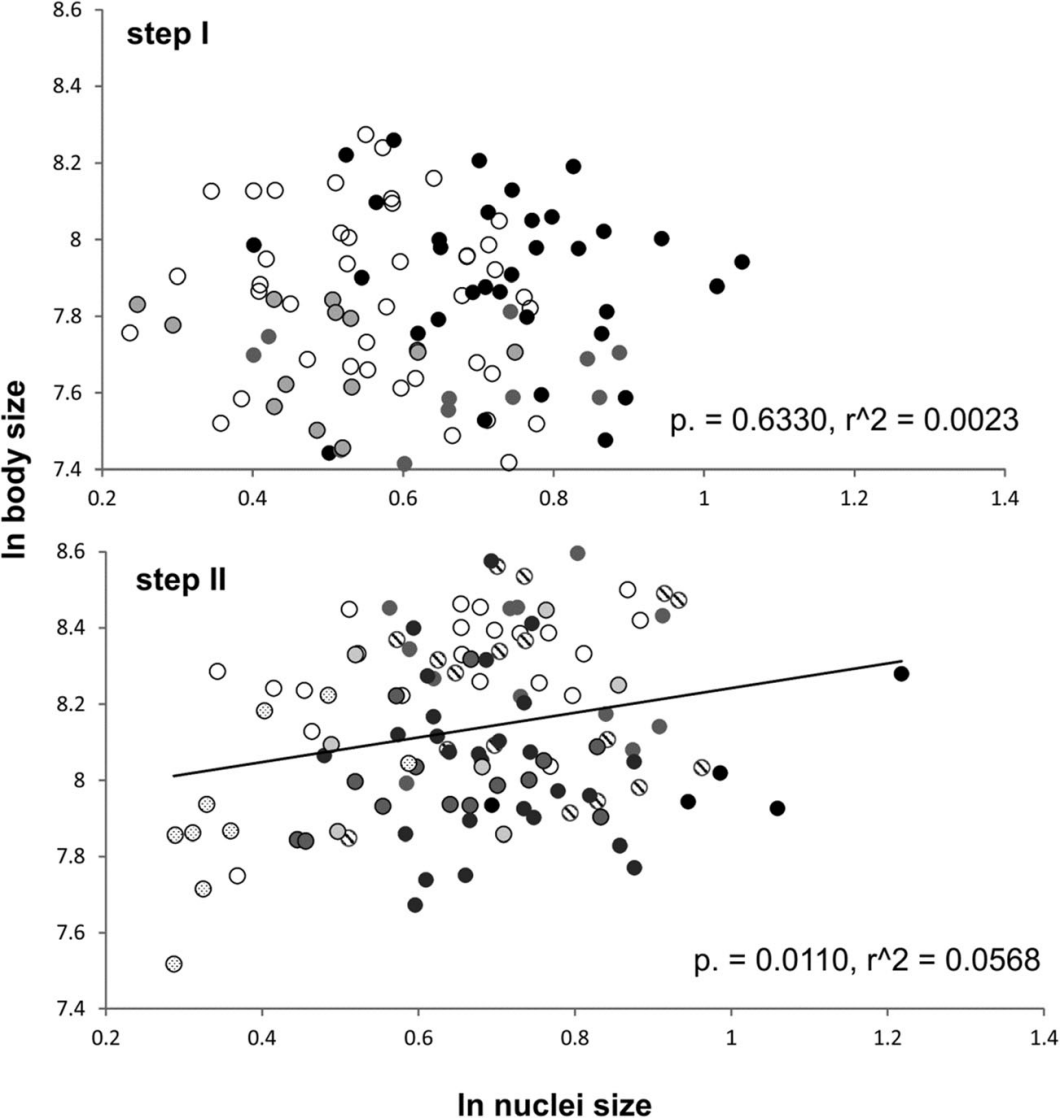
The relationship between body size and cell size in *L. inermis* in step I (*upper panel*) and step II (*lower panel*) conditions (unpublished data; methodology described in Walczyńska et al. 2015a). Different treatments are marked with different symbol colors

We found that the relationship between body size and cell size is stronger in the stressful conditions maintained during step II than in step I (Fig. 5) despite the much smaller sample size. We compared this result with the data from the experiment previously conducted on rotifers (Walczyńska et al. 2015a). In that case, the difference was even more pronounced; in step I, the relationship between the individual body and nucleus size of rotifers was not significant (*N* = 101, *p* = 0.6330, *r*^2^ = 0.0023), while this relationship analyzed using the data from step II was significant (*N* = 113, *p* = 0.0110, *r*^2^ = 0.0568; Fig. 7). Thus, it suggests that under mild conditions, the body/cell size match is relaxed while its strict adjustment switches on under more stressful (= physiologically demanding) conditions. To our knowledge, this is the first observation of the condition-dependent strength of the body/cell size adjustment. Additionally, our results show that similar mechanisms of response to stressful conditions are launched in exemplary eutelic (previous study; rotifers) and non-eutelic (this study; annelids) organisms, which may indicate the superordinate role of cell size over cell number in overcoming harsh temperature/oxygen conditions.

A very subtle and interesting aspect of the body/cell size adjustment in our study is that the effect of a larger body size in the Temp− group in trial 2 diminished after including cell size as a covariate in a model (Fig. 6). This intriguing result confirms the condition-dependent strength of body/cell size correspondence, in this case related to temperature increase vs. decrease. For practical use, this observation also shows that the body/cell size relationship should be taken into account in any laboratory study when a body size response to a given variable is to be evaluated.

An indirect confirmation of the importance of the correspondence between the cell and body size in response to temperature may be found in the report by Jalal et al. (2015). The authors focused on different aspects of the interrelationship between genome, cell, and body size in *Drosophila melanogaster*. They found, among others, that the level of DNA condensation decreased with increasing temperature according to the general models, but only in the case of the whole organisms, while the pattern was opposite for the cell cultures. Thus, the response of *Drosophila* cells from the cell culture differed from the cell response in the whole *Drosophila* organism, possibly because of some superordinate orders from the higher levels of body organization in the latter case. This may be associated with the so-called “supracellular service,” a term raised, for example, in the case of the BMR response in different tissues of mice (Maciak et al. 2014) or the microtubular response to mechanical stress in *Arabidopsis thaliana* (Jacques et al. 2013).

### What is the difference in proximate mechanisms behind the response to increasing and decreasing temperature?

The pattern of the importance of the experienced direction of temperature change of individuals, whether experiencing lower (Temp−) or higher (Temp+) temperature, was observed regardless the fact that both “Temp+” and “Temp−” groups actually consisted of annelids deriving from two different temperatures in step I (18 and 21 vs. 24 and 27 °C, respectively; Fig. 1) and regardless of the lack of differences in size between annelids in these groups. This finding means that the cue toward the direction of temperature change may be more important for ectotherms than the temperature itself. Such a result was previously suggested in a study on the TSR determination in *L. inermis* rotifers (Walczyńska et al. 2015b). Two explanatory mechanisms behind different body/cell size matches in Temp−/Temp+ annelids, as illustrated in Fig. 6, are possible:

- H1—A cue of temperature increase acts differently on body/cell size adjustment than a cue of temperature decrease, a constraint, or
- H2—Under stressful conditions, the ratio of body size to cell size is optimized or canalized (sensu Waddington 1942), an adaptation.

Neither of these hypotheses can be tested here because of the small sample size per experimental treatment. However, some light may be shed by the pattern of population numbers in respective groups (trial 2; 30% O_2_ in Fig. 3). It is clear that the demarcation line for fitness does not go through the Temp−/Temp+ treatment but through a combination of this factor interacting with the temperature/oxygen combination, which would signify the limiting, rather than adaptive, meaning of different body/cell size match in Temp− vs. Temp+ annelids. On the other hand, another one of our results shows that the body/cell size match is stronger in stressful than in mild conditions, regardless of the experimental treatment (Fig. 5), and favors the adaptive meaning. We will refer to this issue below.

The study on the effect of temperature on the structural changes in the genome and their further consequences for cell and body size in ectotherms, conducted by Jalal et al. (2013) on *Daphnia*, may act as an indirect confirmation of H1. The authors found that in the reversal experiment, DNA in the cells of *Daphnia* from 20 °C and incubated at 10 °C showed a considerable decrease in both DNA content and its variation in the cells, while no reverse change was observed in the cells of *Daphnia* from 10 °C and incubated at 20 °C. This result could mean that only a cue toward temperature decrease launched a response in DNA content. In the case of *Daphnia magna*, the optimal temperature is approximately 26–28 °C (Lampert 2006). Therefore, 10 °C seems to be suboptimal for this species. It would be interesting to show how this increasing variation in DNA content is reflected in body size, or whether this higher variation at a more stressful temperature is associated with a stronger link between cell size and body size.

### What is limiting and what is adaptive in the cell and body size relationship?

It was proposed that cells (Czarnoleski et al. 2015; Kozłowski et al. 2003; Szarski 1983; Woods 1999) and bodies (Kozłowski et al. 2004; Kozłowski et al. 2003) decrease in size with increasing temperature. On the other hand, Hessen et al. (2013) proposed the interesting bottom-up perspective, in which the link between genome size and body size is driven by the genome size increase at low temperature, caused by the limitation in enzyme kinetics (Xia 1995) or in the efficiency of protein synthesis (Woods et al. 2003) at lower temperature. From the perspective of our results, these options are not mutually exclusive; it is possible that a temperature increase limits the oxygen transport efficiency, launching the cell size decrease through relevant changes in DNA structure, while a temperature decrease limits the proper genome maintenance, forcing the increase in the amount of DNA, followed by an increase in cell (and body) size. Further tests would demand studies on the genome, cell, and body size response to temperature, changing to both directions and conducted under optimal (mild) and suboptimal (stressful) conditions. The relatively well-known taxon regarding this matter is a widely studied group of crustaceans. For the case of amphipods, it was suggested that larger genomes may be associated with stable environments (Rees et al. 2007). Additionally, the study in Lake Baikal showed that genome size coevolved in this group of animals with body size in response to the selection to different habitats (= lake depth), but what the target of selection was (genome or body) remains unclear (Jeffery et al. 2017). Finally, climate conditions affect the body size composition in the case of both calanoid (Leinaas et al. 2016) and cyclopoid copepods (Rasch and Wyngaard 2006). In the latter case, a small genome size was suggested to be favored in stressful habitats (Rasch and Wyngaard 2006).

Recently, a stimulating discussion has been opened in the literature regarding the distinction between developmental noise and phenotypic plasticity and their consequence for evolution (Ghalambor et al. 2015; Woods 2014; Woods and Wilson 2015). The issue of body/cell size match under stressful conditions seems to be a promising study system for hypothesis testing.
